# Supplementary material for: Characterization of the genome and silk-gland transcriptomes of Darwin’s bark spider (Caerostris darwini)
Source: PLoS One. 2022 Jun 6;17(6):e0268660. doi: 10.1371/journal.pone.0268660 (PMC9170102; doi:10.1371/journal.pone.0268660)
Supplement: S2 Fig — Relative transcript abundance of spidroin targets plus controls across silk gland samples are presented, normalized to leg tissue samples and calculated using the 2-ΔΔCT method. (PDF) [file pone.0268660.s002.pdf]

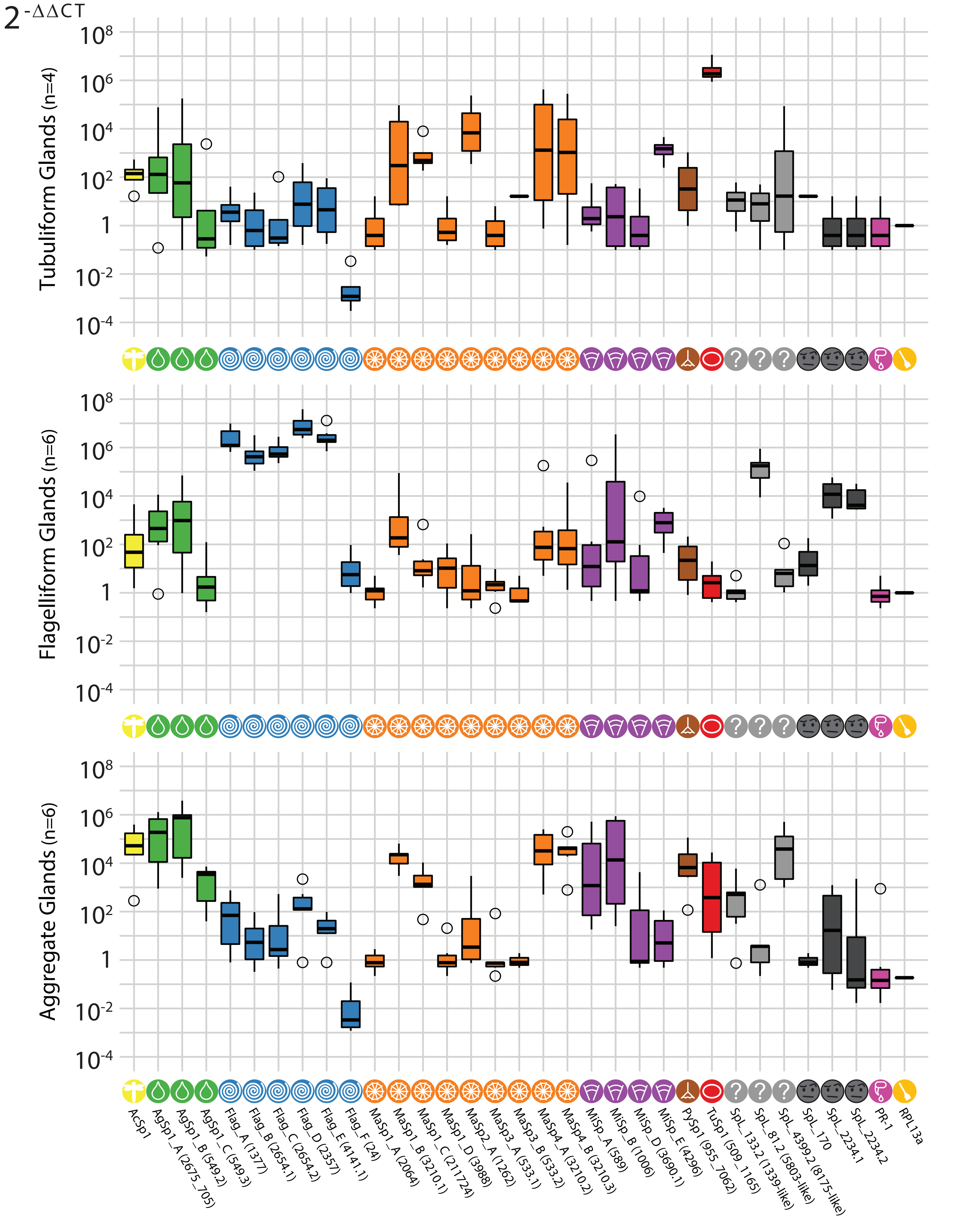

Supplementary Figure 2: qPCR results for spidroins (females) in tubuliform, flagelliform, and aggregate silk glands. Relative transcript abundance of spidroin targets plus controls across silk gland samples are presented, normalized to leg tissue samples and calculated using the 2-ΔΔCT method.
