# Supplementary material for: Characterization of the genome and silk-gland transcriptomes of Darwin’s bark spider (Caerostris darwini)
Source: PLoS One. 2022 Jun 6;17(6):e0268660. doi: 10.1371/journal.pone.0268660 (PMC9170102; doi:10.1371/journal.pone.0268660)
Supplement: S3 Fig — Relative transcript abundance of spidroin targets plus controls across silk gland samples are presented, normalized to leg tissue samples and calculated using the 2-ΔΔCT method. (PDF) [file pone.0268660.s003.pdf]

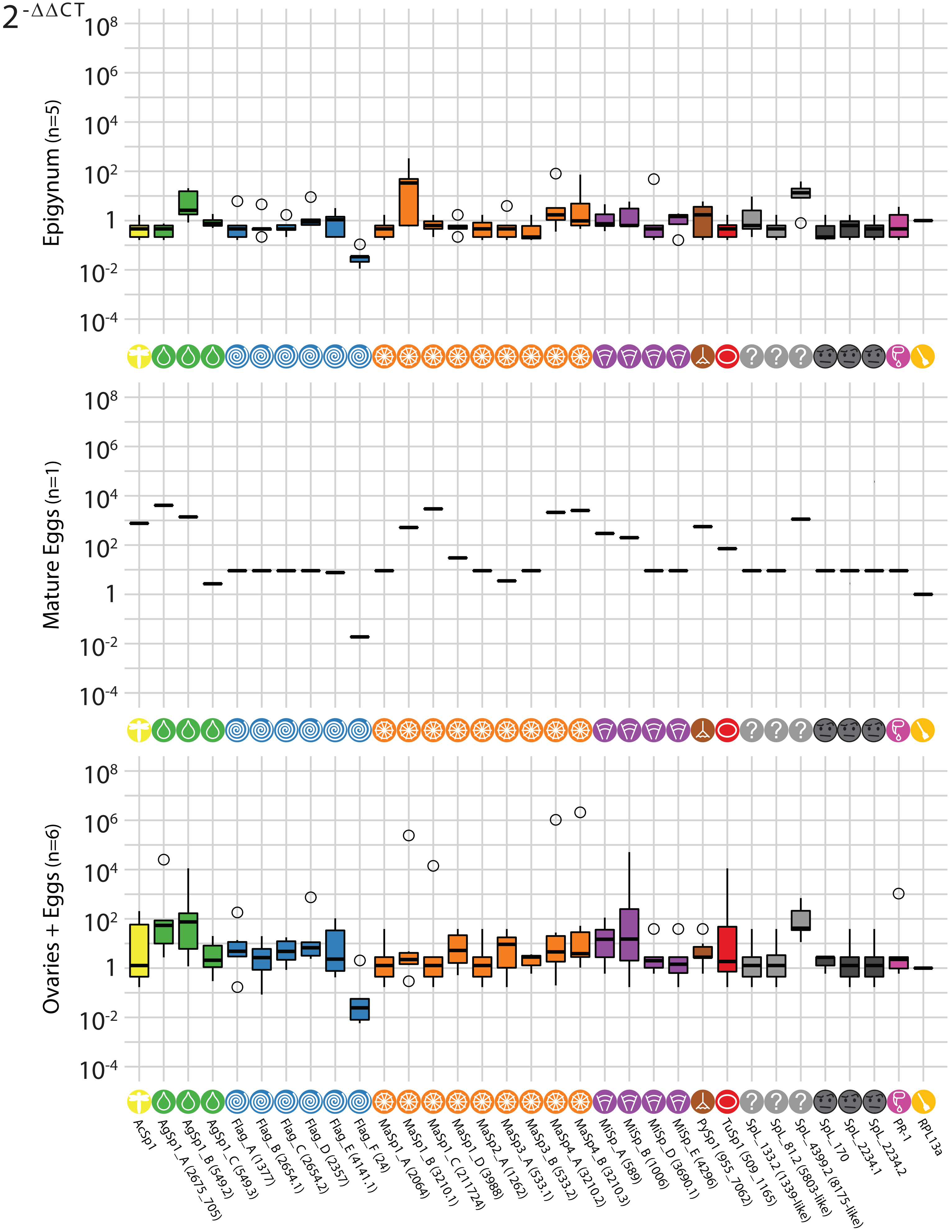

Supplementary Figure 3: qPCR results for spidroins (females) in epigynum, mature eggs, and ovaries + eggs. Relative transcript abundance of spidroin targets plus controls across silk gland samples are presented, normalized to leg tissue samples and calculated using the 2-ΔΔCT method.
